# Molecular signatures of lymph node status by intrinsic subtype: gene expression analysis of primary breast tumors from patients with and without metastatic lymph nodes

**DOI:** 10.1186/s13046-014-0116-3

**Published:** 2014-12-31

**Authors:** Craig D Shriver, Matthew T Hueman, Rachel E Ellsworth

**Affiliations:** Clinical Breast Care Project, Murtha Cancer Center, Walter Reed National Military Medical Center, 8901 Rockville Pike, Bethesda, MD 20889 USA; Clinical Breast Care Project, Murtha Cancer Center, 620 Seventh Street, Windber, PA 15963 USA

**Keywords:** Lymph node status, Subtype, Breast cancer metastasis

## Abstract

**Background:**

Identification of a gene expression signature in primary breast tumors that could classify patients by lymph node status would allow patients to avoid the morbidities of surgical disruption of the lymph nodes. Attempts to identify such a signature have, to date, been unsuccessful. Because breast tumor subtypes have unique molecular characteristics and different sites of metastasis, molecular signatures for lymph node involvement may vary by subtype.

**Methods:**

Gene expression data was generated from HG U133A 2.0 arrays for 135 node positive and 210 node negative primary breast tumors. Intrinsic subtype was assigned using the BreastPRS. Differential gene expression analysis was performed using one-way ANOVA using lymph node status as the variable with a False-discovery rate <0.05, to define significance.

**Results:**

Luminal A tumors were most common (51%) followed by basal-like (27%), HER2-enriched (14%) luminal B (7%) and normal-like (1%). Basal-like and luminal A tumors were less likely to have metastatic lymph nodes (35% and 37%, respectively) compared to luminal B or HER2-enriched (52% and 51%, respectively). No differentially expressed genes associated with lymph node status were detected when all tumors were considered together or within each subtype.

**Conclusions:**

Gene expression patterns from the primary tumor are not able to stratify patients by lymph node status. Although the primary breast tumor may influence tumor cell dissemination, once metastatic cells enter the lymphatics, it is likely that characteristics of the lymph node microenvironment, such as establishment of a pre-metastatic niche and release of pro-survival factors, determine which cells are able to colonize. The inability to utilize molecular profiles from the primary tumor to determine lymph node status suggest that other avenues of investigation, such as how systemic factors including diminished immune response or genetic susceptibility contribute to metastasis, may be critical in the development of tools for non-surgical assessment of lymph node status with a corresponding reduction in downstream sequelae associated with disruption of the lymphatics.

## Background

Surgical treatment for patients with breast cancer is constantly changing [1]. The radical mastectomy, which removed the breast, underlying chest muscle and axillary lymph nodes has been supplanted by less aggressive approaches such as lumpectomy, and complete removal of the axillary lymph nodes has been replaced by sentinel lymph node biopsy (SLNB) [2,3]. Recent results from the ACOSOG Z0011 trial demonstrate that SLNB performed without follow up axillary dissection is reasonable for patients with early-stage, lymph node positive breast cancer [4].

Although SLNB is associated with lower morbidities, surgical disruption of the lymphatic system can result in serious side effects, including numbness, decreased mobility and lymphedema, significantly impacting the quality of life of breast cancer patients. For example, lymphedema can result in pain, decreased functional ability, cosmetic deformities and psychological stress [5] and is estimated to affect 10-20% of breast cancer survivors [6]. In addition, SLNB is associated with a false negative rate of 8-10% [7,8]. Development of a signature that effectively discriminates patients by lymph node status could stratify patients into those needing surgical evaluation of the lymph nodes for prognostic purposes from those at low-risk of metastasis who may be spared possible serious side effects as well as identify those patients misdiagnosed with negative lymph node status after SLNB, who may in fact benefit from more aggressive treatment.

Although a few studies have identified genes or proteins expressed in primary tumors that differ in expression levels based on lymph node status [9-14], other studies failed to validate these results and/or found that molecular profiling of primary tumors cannot effectively classify patients by lymph node status [15-18]. Inability to identify a signature of lymph node metastasis may be attributable to heterogeneity within primary breast tumors associated with intrinsic subtypes. Breast tumors can be classified into subtypes, including luminal A, luminal B, HER2 positive and basal-like, based on different patterns of gene expression. Molecular heterogeneity within tumor subtypes may also preclude the identification of a single signature of metastasis. Breast tumors can be classified by their intrinsic subtypes, including luminal A, luminal B, HER2 positive and basal-like, based on different patterns of gene expression [19,20]. These subtypes have been associated with differences in preferential sites of metastasis; for example, bone is the most common site of metastasis for luminal A tumors while brain is most common for basal-like tumors [21]. Because breast tumor intrinsic subtypes have unique molecular characteristics and different sites of metastasis, gene expression patterns for lymph node involvement may vary by subtype, thus gene expression data from primary breast tumors with and without lymph node metastases was evaluated by intrinsic subtype to identify subtype-specific molecular signatures associated with lymph node status.

## Methods

For inclusion in the Clinical Breast Care Project, all patients met the following eligibility criteria: 1) adult over the age of 18 years, 2) mentally competent and willing to provide informed consent, and 3) presenting to the breast centers with evidence of possible breast cancer. Tissue and blood samples were collected with approval from the Walter Reed National Military Medical Center Human Use Committee and Institutional Review Board. All subjects voluntarily agreed to participate and gave written informed consent.

Positive lymph node status was defined as having micrometastatic (>0.2 mm but ≤ 2.0 mm) or metastatic (>2.0 mm) lymph node tumors; negative lymph node status was defined as lymph nodes with isolated tumor cells (≤0.2 mm) or no detectable tumor cells. Patients who underwent neoadjuvant therapy and those diagnosed with stage IV breast cancer were not included in this study. Tissue was collected from patients undergoing surgical procedures, including lumpectomy or mastectomy. Within 5–15 minutes of surgical removal, breast tissue was taken on crushed, wet ice to the pathology laboratory where a licensed pathologist or pathologists’ assistant performed routine pathology analyses. Two to five serial sections (8 μm thick) were cut, mounted, stained and laser microdissected as previously described [15]. Slide preparation, staining and cutting were performed within 15 minutes to preserve RNA integrity. RNA for microarray analysis was processed as previously described [22]. Labeled RNA was hybridized to HG U133A 2.0 arrays (Affymetrix, Santa Clara, CA) according to manufacturer’s protocols.

Intrinsic breast subtype was assigned to each tumor specimen using the BreastPRS™ (Signal Genetics, New York, NY) as previously described [23,24]. Samples were classified as one of five subtypes: luminal A, luminal B, HER2-enriched, basal-like and normal-like. To determine whether genes were differentially expressed by lymph node status by subtype, gene expression data was imported into Partek® Genomics Suite 6.6 (Partek, Inc, St Louis, MO) and analyzed as previously described [15], using a false discovery rate (FDR) < 0.05 to define significance. Additional analyses were performed using an unadjusted P-value to identify any genes differentially expressed between primary breast tumors without and without lymph node metastasis.

## Results

### Patient and tumor characteristics

**Figure 1:**
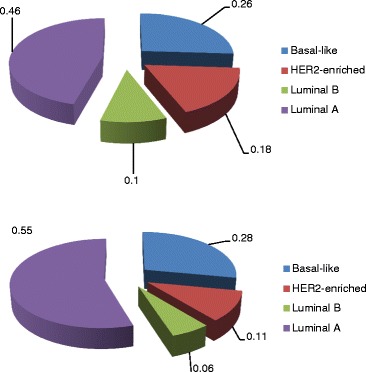
**Distribution of intrinsic subtypes by lymph node status.** Subtype frequencies were significantly different (P < 0.05) between groups, with lymph node negative tumors (top chart) having a higher frequency of luminal A tumors while node positive tumors (bottom chart) had higher frequencies of luminal B and HER2-enriched tumors. Frequencies of normal-like tumors were <1.0% for each group.

Gene expression data was available for 345 tumors: 210 lymph node negative and 135 lymph node positive. Ethnicity, age at diagnosis and tumor grade did not differ significantly by lymph node status, however, patients diagnosed with positive lymph nodes (7%) were significantly more likely (P < 0.05) to die of disease than those with negative lymph node status (2%) at diagnosis. The most common intrinsic subtype was luminal A (51%), followed by basal-like (27%), HER2-enriched (13%), luminal B (7%) and normal-like (1%) (Figure 1). When stratified by lymph node status, subtype distribution was significantly different, with higher frequencies of HER2-enriched and luminal B subtypes in the lymph node positive group.

### Gene expression differences in primary breast tumors by lymph node status

**Figure 2:**
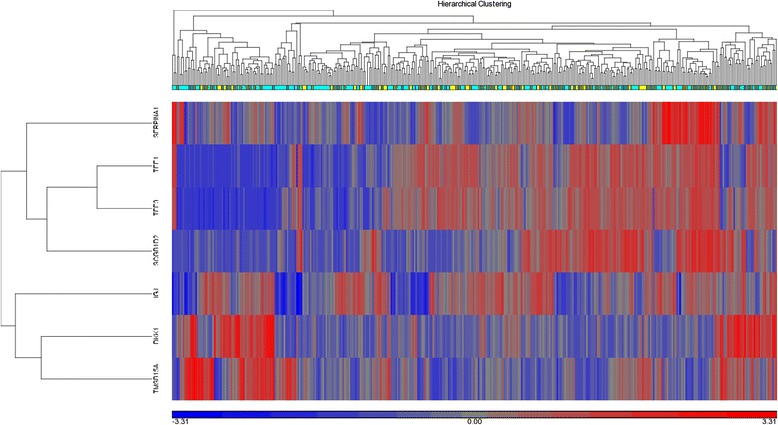
**Heat map of node negative and node positive breast tumors.** Seven genes (DKK1, IGJ, SCGB1D2, SERPINA1, TFF1, TFF3 and TMSB15A) were found to be differentially expressed using an unadjusted P-value <0.05 that does not correct for multiple testing. These seven genes were unable to effectively classify tumors by lymph node status. Tumors from node negative patients are represented by light blue squares and node positive patients represented by yellow squares.

Principal component analysis (PCA) did not cluster the tumors based on lymph node status. Gene expression analysis of all tumors failed to identify any differentially expressed genes using FDR < 0.05, with any fold difference. To determine whether any genes could classify tumors by lymph node status, an unadjusted P-value was used; seven differentially expressed genes were identified, however, these genes were not able to effectively cluster primary tumor specimens (Figure 2). When data were evaluated by size of metastasis (isolated tumor cells, micrometastsis or metastasis) or number of positive nodes (1–3, 4–9 or ≥10) no significant differences were detected.

### Subtype-specific gene expression differences by lymph node status

Although PCA did not cluster samples by lymph node status, it was effective in clustering samples by subtype, underscoring the significantly different molecular characteristics between tumor subtypes. To determine whether a signature(s) of lymph node metastasis was detectable within individual subtypes, data was evaluated for luminal A (n = 177), luminal B (n = 25), HER2-enriched (n = 47) and basal-like (n = 94) lymph node negative and lymph node positive tumors; normal-like tumors (n = 2) were not evaluated. No significant gene expression differences were detected within any of the subtypes.

## Discussion

Identification of a gene expression signature predictive of lymph node metastasis would further the evolution of clinical treatment for breast cancer by allowing for the determination of nodal status based on molecular characteristics of the primary breast tumor, allowing women to be spared surgical disruption of the axillary lymph nodes. In addition, a molecular signature would provide a tool to determine lymph node status in those patients not eligible for SLNB and as well as to reduce the false negative rate of SLNB. No differentially expressed genes were identified in node negative compared to node positive tumors either when primary breast tumors were considered as a whole, or within the four intrinsic subtypes luminal A, luminal B, HER2-enriched or basal-like.

The inability to identify a signature of lymph node metastasis underscores the complexity of the metastatic process. Although thousands of cells are disseminated from a primary breast tumor, growth of metastatic tumors requires tumor cells to successfully reach the secondary site, escape senescence and survive and proliferate within a foreign environment [25,26]. For example, gene expression patterns in primary breast tumors differ significantly from matched metastatic lymph node tumors, with genes expressed in the primary tumor favoring cellular dissemination, while those in metastatic lymph node tumors are involved in cellular proliferation and survival [27]. In addition, tumor cells are not self-reliant but rather depend on a complex interaction with the microenvironment [28]. For example, many signatures of poor prognosis or of metastasis include the expression of stromal genes. Recent data from our laboratory demonstrated that gene expression profiles differed in lymph nodes harboring metastatic breast tumors when compared to negative lymph node tissues and that these differences created an immunotolerant environment promoting cellular proliferation and the mesenchymal-epithelial transition, all of which favors tumor growth [29]. Thus, consideration of only the tumor epithelial component may fail to capture the full metastatic potential of a primary tumor.

In addition, tumor heterogeneity may confound the ability to identify a molecular signature of lymph node metastasis. Primary tumors demonstrate significant heterogeneity at the molecular level include expression of prognostic biomarkers such as ER, PR and HER2, chromosomal alterations and DNA mutations [30-34]. Evaluation of protein expression within primary tumors using technologies such as reverse phase protein arrays also demonstrates intratumoral heterogeneity with a mean coefficient of variation of 31% within primary tumors [35]. In this study, laser microdissection was utilized to enrich for tumor epithelial cells and reduce contamination by stromal cells; however, tumor regions isolated for this study may contain cells with heterogeneous molecular profiles and/or different levels of metastatic potential, thus diluting the ability to detect gene expression differences associated with metastasis to the lymph nodes.

In addition to the contribution of the microenvironment to successful metastatic colonization, systemic factors, such as inherent host susceptibility may affect the metastatic process. Decreased immunosurveillance and an increased pro-inflammatory response were characteristics of lymph nodes harboring metastatic breast tumors [29]; what remains unknown is whether these alterations in immune response are local or systemic. Studies in mouse models suggest that there is a genetic susceptibility to metastasis as out-crossing of a highly metastatic mouse to a variety of inbred mouse strains resulted in significant variability in the propensity to metastasize [36]; follow-up studies in humans validated the roles of SIPA1 and RRP1B as metastasis susceptibility genes [37]. Thus, the ability to successfully metastasize may include systemic as well as tumor/stromal factors.

This study does have limitations. Although gene expression data was available from 345 primary breast tumors, only 25 (7%) were of the luminal B subtype. Thus, a gene signature for lymph node metastasis within the primary tumor may be present, although such a signature was undetected within the other subtypes. In addition, only RNA was evaluated in this study: DNA alterations or protein profiles may be effective in discriminating primary tumors by lymph node status. For example, copy number alterations and were detected at significantly higher levels and GSTP1 and RAR-beta2 were more likely to be hypermethylated in primary tumors with metastatic lymph nodes compared to those without [38,39], although neither of these genes demonstrated differential expression within our dataset. In addition, protein signatures based on differentially expressed protein peaks or proteins have been identified [40,41], although both protein signatures included pathological characteristics, such as tumor size, in their predictive model that are known to be predictive of lymph node metastasis, thus it is not clear whether these signatures have independent prognostic value.

## Conclusions

Significant differences in gene expression levels are not detectable in lymph node positive compared to lymph node negative tumors, even within intrinsic subtypes. The inability to identify a signature of metastasis reflects the complexities underlying the metastatic process, in which tumor cells grow and survive only in collaboration with the microenvironment and against a pro-metastatic genetic background. Because molecular profiles from primary tumors cannot predict nodal status, other avenues of investigation, such as how diminished immune response or genetic susceptibility contribute to metastasis, must be pursued to further the evolution of clinical care of breast cancer patients.
